# Impact of diagnosis-to-treatment interval on the outcome of patients with acute myeloid leukemia

**DOI:** 10.1007/s00277-026-07052-7

**Published:** 2026-05-09

**Authors:** Samah Nassereddine, Yuan Feng, Jordan Selep, Firas El Chaer, Leah Wells, Ramey Elsarrag, Kimberly Doucette, Vanya Aggarwal, Lacey Williams, Imad Tabbara, Shanshan Liu, Guoquing Diao, Mary-Elizabeth M. Percival, Catherine Lai

**Affiliations:** 1 GW Cancer Center, George Washington University, Washington, DC USA; 2 Department of Biostatistics and Bioinformatics, Milken Institute School of Public Health, George Washington University, Washington, DC USA; 3 Division of Hematology and Oncology, Department of Medicine, University of Virginia, Charlottesville, VA USA; 4 Department of Medicine, Georgetown University, Washington, DC USA; 5 Clinical Research Division, Fred Hutchinson Cancer Center, Seattle, WA USA; 6 Division of Hematology and Oncology, Abramson Cancer Center, University of Pennsylvania, Philadelphia, PA USA

**Keywords:** Acute myeloid leukemia, Diagnosis-to-treatment interval, Induction chemotherapy, Molecular testing, Risk stratification, Targeted therapy, Precision medicine, Treatment outcomes

## Abstract

Acute myeloid leukemia (AML) is considered an oncologic emergency, yet the optimal timing for treatment initiation remains uncertain. We conducted a multi-institutional retrospective study of 698 adults with newly diagnosed AML presenting to four academic centers across the United States. Diagnosis-to-treatment intervals (DTI) were categorized as < 5 days, 5–10 days, and > 10 days. Outcomes were analyzed using multivariable models adjusting for age, treatment intensity, ELN 2017 risk classification, and white blood cell count. Among younger patients, DTI was not associated with differences in survival outcomes. In contrast, older patients demonstrated improved survival with delayed treatment (DTI > 10 days), particularly those with lower white blood cell counts. No adverse effects from treatment delay were observed in younger cohorts. This retrospective study showed that prolonged DTI is associated with improved survival in older adults with newly diagnosed AML, challenging the traditional assumption that immediate therapy universally improves outcomes. These findings underscore the importance of individualized treatment timing in the era of precision oncology.

**Supplementary Information:**

The online version contains supplementary material available at 10.1007/s00277-026-07052-7.

## Introduction

The diagnosis of acute myeloid leukemia (AML) is an oncologic emergency that often necessitates prompt initiation of therapy. The optimal timing of therapy initiation has long been debated, and current guidelines do not provide specific recommendations regarding the diagnosis-to-treatment interval (DTI) in patients with AML. Historically, prompt initiation of induction chemotherapy was considered crucial to mitigate disease-related morbidity and mortality, particularly among younger patients with proliferative AML [1].

In recent years, a large retrospective study from Germany showed that delayed DTI was not associated with worse outcome [2]. In a similar pattern, another retrospective study focused on patients treated with hypomethylating agent and venetoclax, did not show harmful effect from delaying treatment [3]. These studies challenge the historic emphasis on urgency in initiating leukemia directed treatment and put in question our knowledge about the factors that could impact our patients outcome. Whether delaying treatment offers time to better optimize treatment decision based on molecular basis or whether stabilizing the patients from infection or DIC are crucial prior to initiating leukemia directed therapy. This underscores the updated revision of the World Health Organization (WHO) of myeloid neoplasm and the International Consensus Classification (ICC) systems, where molecular characterization has become increasingly integral to AML diagnosis and risk stratification [4–6].The expanding molecular landscape of AML informs therapeutic decision-making and disease monitoring. Over the past decade, the introduction of targeted therapies for actionable mutations has transformed AML management, highlighting the growing importance of genomic profiling in guiding treatment decisions and improving clinical outcomes [7–10].

The BEAT AML Master clinical trial, the first prospective precision medicine trial in hematologic malignancies, demonstrated superior outcomes when patients with actionable mutations received targeted therapy rather than standard of care [11]. In addition, delaying therapy to await molecular testing results was found to be safe in patients without evidence of hyperleukocytosis.

In routine practice, however, the turnaround time for comprehensive molecular profiling can be prolonged, typically requiring 7 to 14 days for completion. Although enrollment in clinical trials may expedite access to results and targeted agents, most patients are treated in real-world settings with standard of care regimens.

To address the clinical uncertainty surrounding the timing of therapy initiation, we conducted a multi-institutional retrospective study across four U.S. academic centers to address the impact of DTI on outcomes in newly diagnosed AML. Our study aimed to define the optimal treatment window in the contemporary era of precision oncology, where molecular testing increasingly guides therapeutic selection and clinical decision-making.

## Methods

We retrospectively collected data on 698 adult patients (aged ≥ 18 years) with newly diagnosed AML treated at four academic cancer centers between 2010 and 2021: the GW Cancer Center, Georgetown University Hospital, University of Virginia, and University of Washington/Fred Hutchinson Cancer Center. Patients with acute promyelocytic leukemia and those who elected to receive treatment at other institutions were excluded. Baseline demographic and clinical characteristics were obtained through detailed chart review and included age, sex, race, smoking status, comorbidities, and disease characteristics such as presenting white blood cell (WBC) count, bone marrow morphology, cytogenetic and fluorescence in situ hybridization (FISH) results, and next-generation sequencing (NGS) findings when available. Treatment-related variables included regimen intensity (low vs. high) and time from diagnosis to therapy initiation. Low-intensity therapy was defined as treatment with a hypomethylating agent (HMA) alone, HMA combined with venetoclax, or low-dose cytarabine (LoDAC) with or without venetoclax. High-intensity therapy was defined as conventional intensive induction chemotherapy (i.e., “7 + 3” or a high-dose cytarabine containing regimen). Pre-phase cytoreductive measures such as hydroxyurea or short course cytarabine used solely for leukocytosis control were not considered definitive therapy and were not used to define treatment initiation. Date of diagnosis was defined as the date of first bone marrow biopsy confirming AML or, in cases diagnosed by peripheral blood findings alone, the date of first documentation of ≥ 20% circulating blasts consistent with AML. For referred patients, the original diagnostic date from the referring institution was used when available. Treatment initiation date was defined as the first administration of definitive AML-directed therapy at the treating center. Institutional review board approval and data-sharing agreements were obtained at all participating sites.

The primary outcome was overall survival (OS), defined as the time from AML diagnosis to death from any cause or last follow-up for censored patients. Patients were categorized into three DTI groups: less than five days (< 1–5), six to ten days (6–10), and more than ten days (> 10).

A landmark analysis was performed to evaluate the association between DTI category and OS and to mitigate immortal time bias. Patients who died or were censored within the first 10 days of diagnosis were excluded. For the remaining cohort, follow-up time was re-set at the landmark, such that time zero (t = 0) corresponded to day 10 post-diagnosis, and OS was measured from the landmark to death or censoring.

Cox proportional hazards regression models were used to evaluate the association between DTI and OS. The < 1–5 day category served as the reference group. Multivariable models were adjusted for clinically relevant covariates, including age (≤ 65 vs. >65 years), baseline WBC count (≤ 100 × 10^9^/L vs. >100 × 10^9^/L), 2017 European Leukemia Net (ELN) risk category (favorable, intermediate, or adverse), and treatment intensity (high vs. low). Interaction terms between DTI and both age and WBC categories were included in to assess whether the effect of DTI on OS differed across subgroups.

Hazard ratios (HRs) with corresponding 95% confidence intervals (CIs) were estimated for all comparisons. Linear contrasts of model coefficients were constructed to derive HRs and 95% CIs for specific subgroup analyses (DTI effects within age or WBC strata and pairwise comparisons between non-reference DTI categories. Confidence intervals were calculated using the Delta method, with log-transformed limits subsequently exponentiated. The proportional hazards assumption was verified by examining the Schoenfeld residuals and associated plots, with no significant violations observed. For analyses involving multiple contrasts, p-values were adjusted using the single-step method based on the multivariate normal distribution of contrasts to control the family-wise error rate. All analyses were performed using the R Statistical Software. Survival analyses were conducted using the *survival* package, and multiple comparison adjustments were implemented using the *multcomp* package. A two-sided p-value < 0.05 was considered statistically significant.

## Results

A total of 698 patients across four academic centers were included in the analysis assessing the impact of the DTI on outcomes in AML. Patients were categorized into three DTI groups: <1–5 days (*n* = 324), 6–10 days (*n* = 117), and > 10 days(*n* = 257). Baseline characteristics are summarized in Table 1, and characteristics of the 35 patients excluded by the landmark approach are summarized in Supplementary Table S1. Patients with delayed therapy (> 10 days) were older (median 66.0 years, interquartile range [IQR] 57–74) compared with those treated within 1–5 days (median 60.5 years, IQR 49–70) and presented with lower white blood cell (WBC) counts (mean 15.0 vs. 52.9 × 10³/µL). Elevated WBC counts (> 100 × 10³/µL) were most common among patients treated within five days (19.1%), compared with 5.1% in the 6–10 day group and 1.9% in the > 10 day group (Fisher’s exact test, *p* < 0.001). Adverse ELN risk was more frequent in the > 10-day group (46.7%) than in the < 1–5-day group (38.3%, Fisher’s exact test, *p* = 0.005). High-intensity induction therapy was more often administered to patients with early DTI (77.5% for < 1–5 days vs. 57.2% for > 10 days; Fisher’s exact test, *p* < 0.001).

**Table 1:**
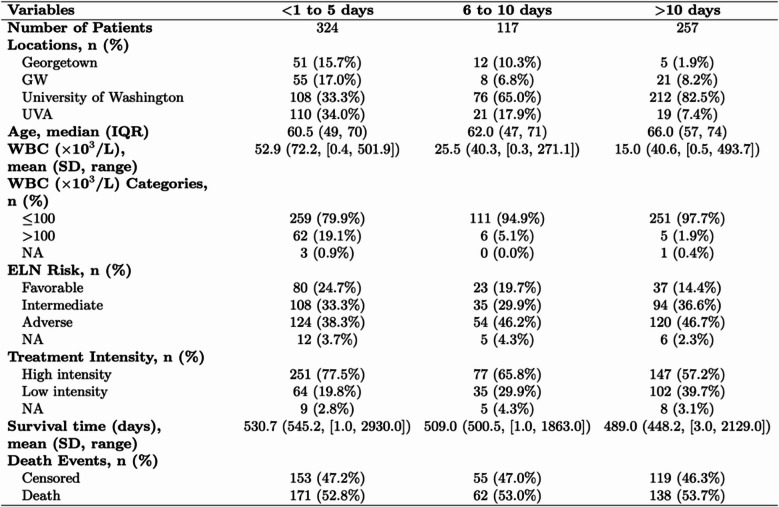
Patient Characteristics by Diagnosis-to-Treatment (DTI) Following Landmark Analysis toAddress Immortal Time Bias; Abbreviations: IQR, interquartile range

**Fig. 1:**
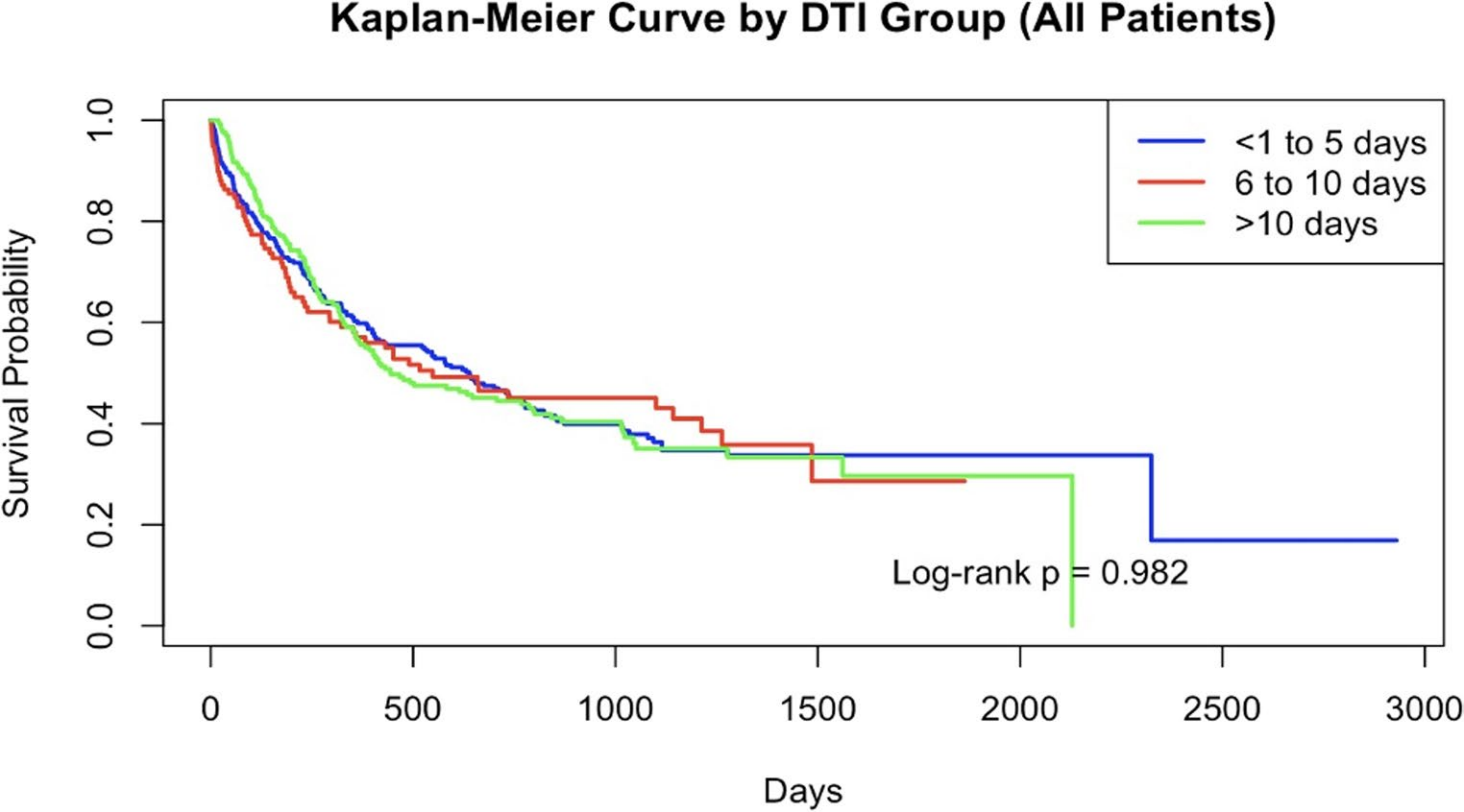
Kaplan-Meier survival curves for AML patients with WBC count < 100,000/uL, stratified byDTI

Kaplan–Meier survival curves demonstrated substantial overlap across DTI categories, with no significant difference by log-rank test (*p* = 0.982; WBC ≤ 100 × 10³/µL subgroup *p* = 0.949) (Fig. 1). Similarly, unadjusted Cox analyses revealed no significant association between DTI and survival, with hazard ratio of 1.02 (95% CI: 0.77–1.37) for the 6–10 day and 1.02 (95% CI: 0.81–1.27) for the > 10-day groups versus the < 1–5 day reference.

After multivariable adjustment for age, WBC count, ELN risk, and treatment intensity (Table 2), a prolonged DTI (> 10 days) was associated with improved survival, corresponding to a 32% reduction in mortality risk compared with early treatment (HR 0.68, 95% CI: 0.53–0.87,*p* = 0.003). Subgroup analyses (Table 3) revelated that this benefit was primarily observed among older patients (> 65 years) with WBC ≤ 100 × 10³/µL, in whom a DTI > 10 days conferred a 38% lower mortality risk compared with treatment within five days (HR 0.62, 95% CI: 0.39–0.99, adjusted *p* = 0.040). Within this same subgroup, outcomes were also superior for patients treated after > 10 days compared with those treated within 6–10 days (HR 0.53, 95% CI: 0.30–0.95, adjusted *p* = 0.024). No statistically significant survival differences by DTI were observed among younger patients (≤ 65 years) or among those with WBC > 100 × 10³/µL after adjustment for covariates. A location-stratified sensitivity analysis for the primary multivariable model is shown in Supplementary Table  S2 .

**Table 2:**
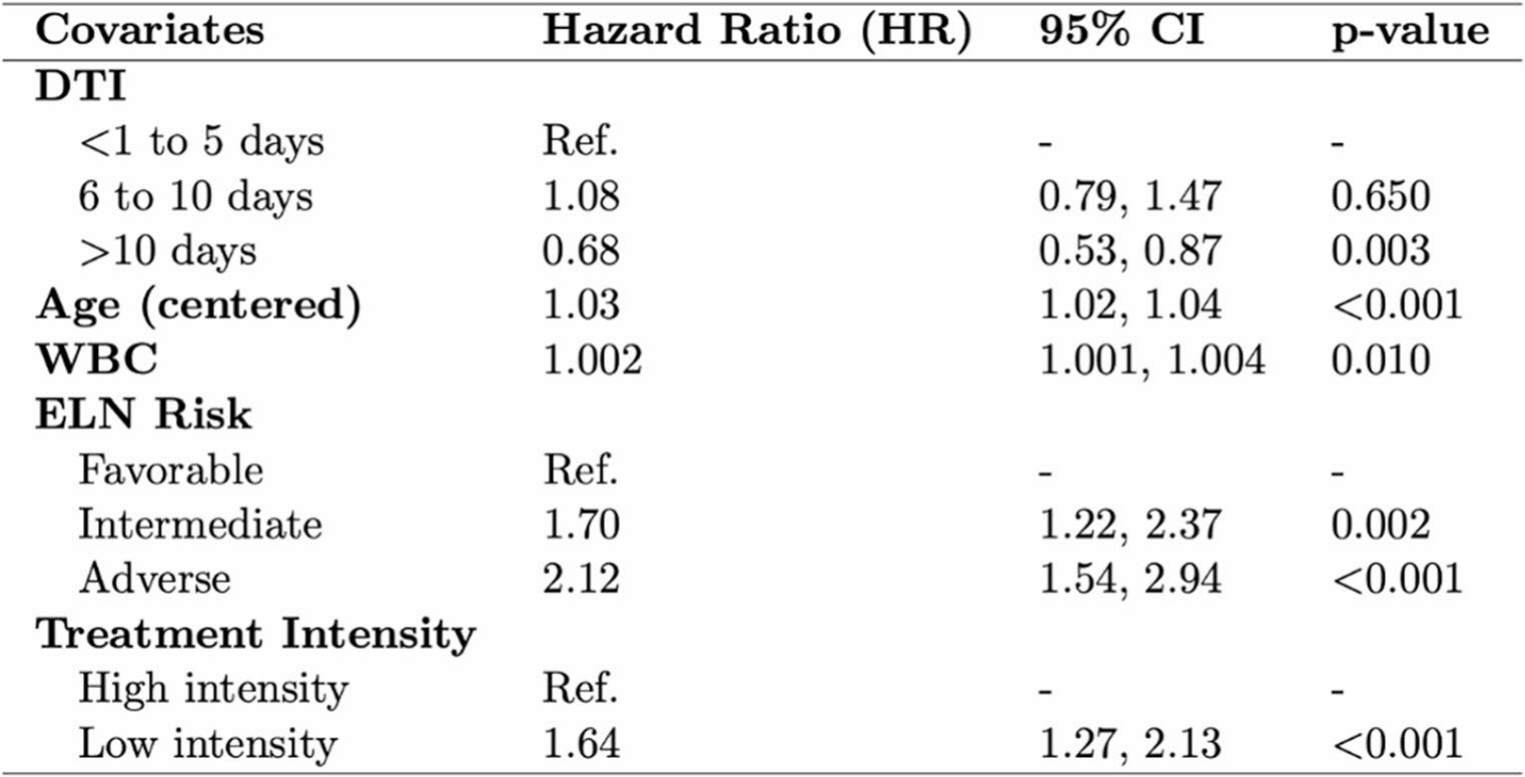
Cox proportional hazards model for AML survival adjusting for age, WBC, ELN risk classification, and treatment intensity

**Table 3:**
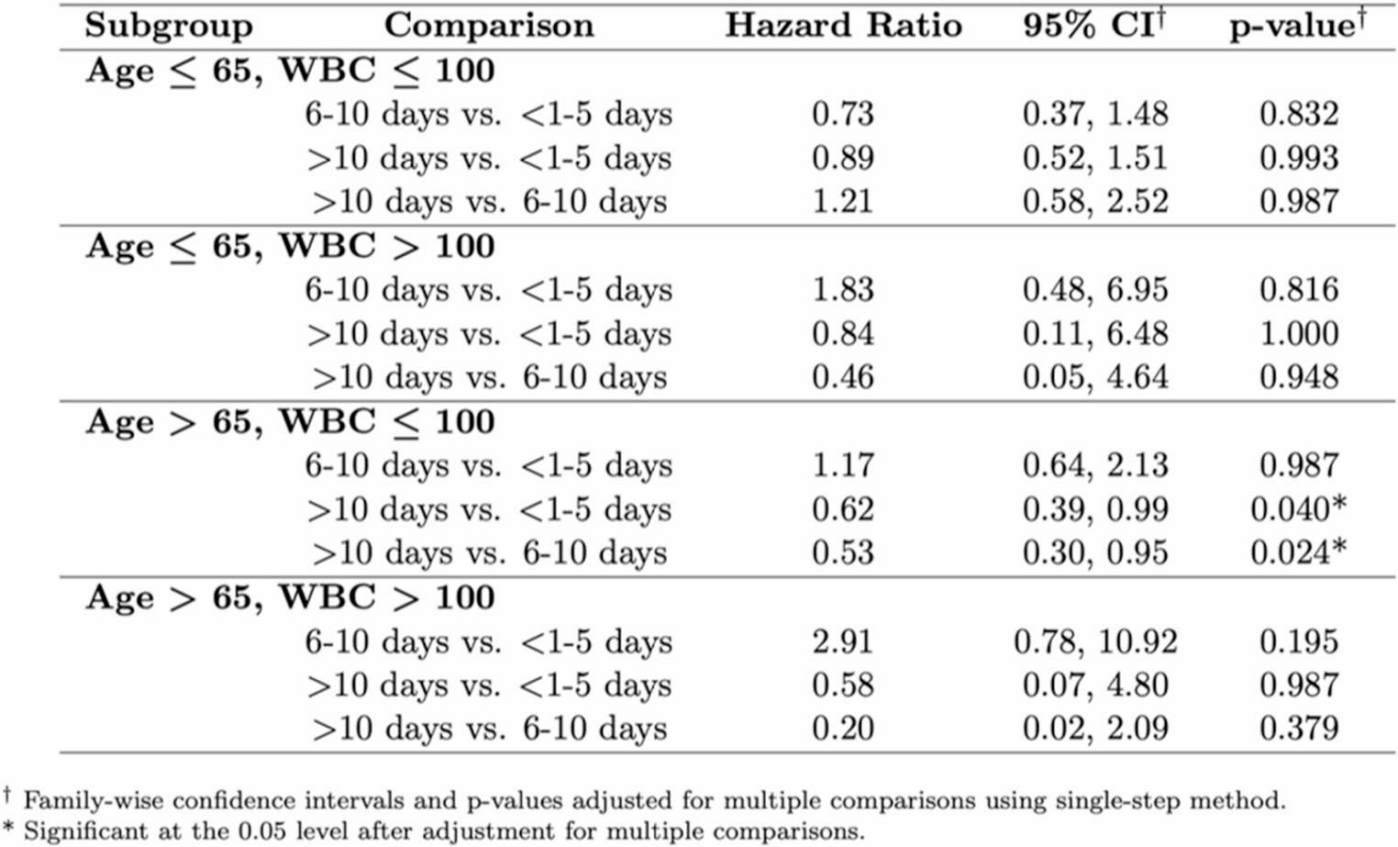
Effect of DTI on survival by age and WBC subgroups with multiple testing adjustment (All hospitals)

Independent of DTI, several factors were significantly associated with survival. Adverse predictors included older age (per year; HR 1.03, 95% CI 1.02–1.04), higher WBC count (per 10³/µL; HR 1.002, 95% CI 1.001–1.004), intermediate ELN risk (HR 1.70, 95% CI 1.22–2.37), adverse ELN risk (HR 2.12, 95% CI 1.54–2.94), and low-intensity therapy vs. high-intensity (HR 1.64, 95% CI 1.27–2.13) (Table 2). These associations were similar in interaction models, with older age (> 65 vs. ≤ 65) (HR 2.14, 95% CI 1.54–2.98), intermediate ELN risk (HR 1.76, 95% CI 1.26–2.46), adverse ELN risk (HR 2.20, 95% CI 1.59–3.05), and low-intensity therapy (HR 1.81, 95% CI 1.41–2.31) continuing to predict inferior outcomes (Table 4). The corresponding location-stratified sensitivity analysis for the interaction model is presented in Supplementary Table  S3 . The only factor independently associated with improved OS was a prolonged DTI > 10 days, an effect restricted to older patients with lower WBC counts, as noted above. We also examined whether DTI distribution differed across calendar periods (2010–2015 vs. 2016–2021). A 3 × 2 contingency analysis using Monte Carlo Fisher testing showed no significant temporal shift in DTI distribution (*p* = 0.292). The distribution by diagnosis period is shown in Supplementary Table S4, with the corresponding Fisher’s exact test in Supplementary Table S5.

**Table 4:**
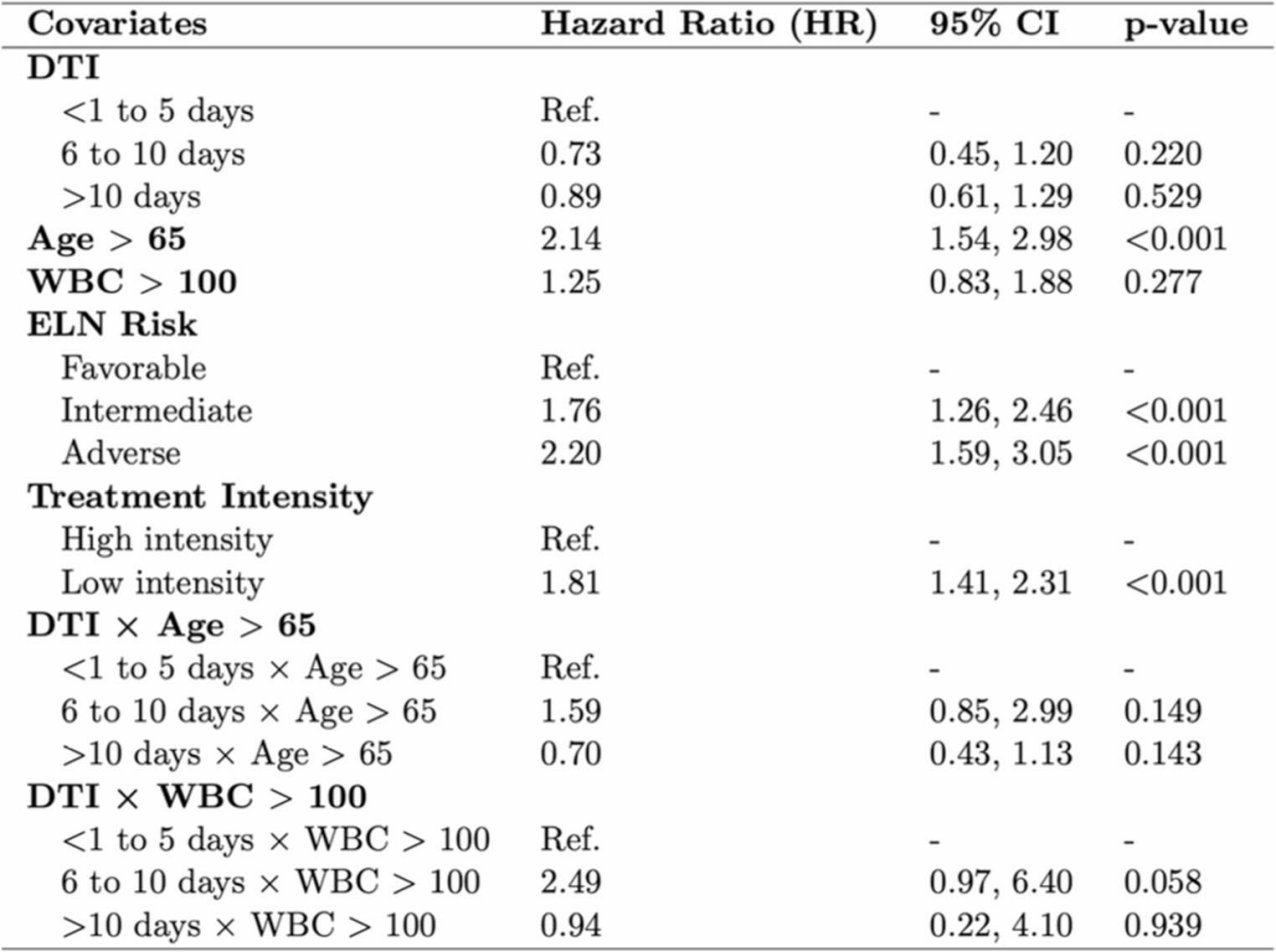
Cox proportional hazards model with age and WBC interaction terms (All hospitals)

**Fig. 2:**
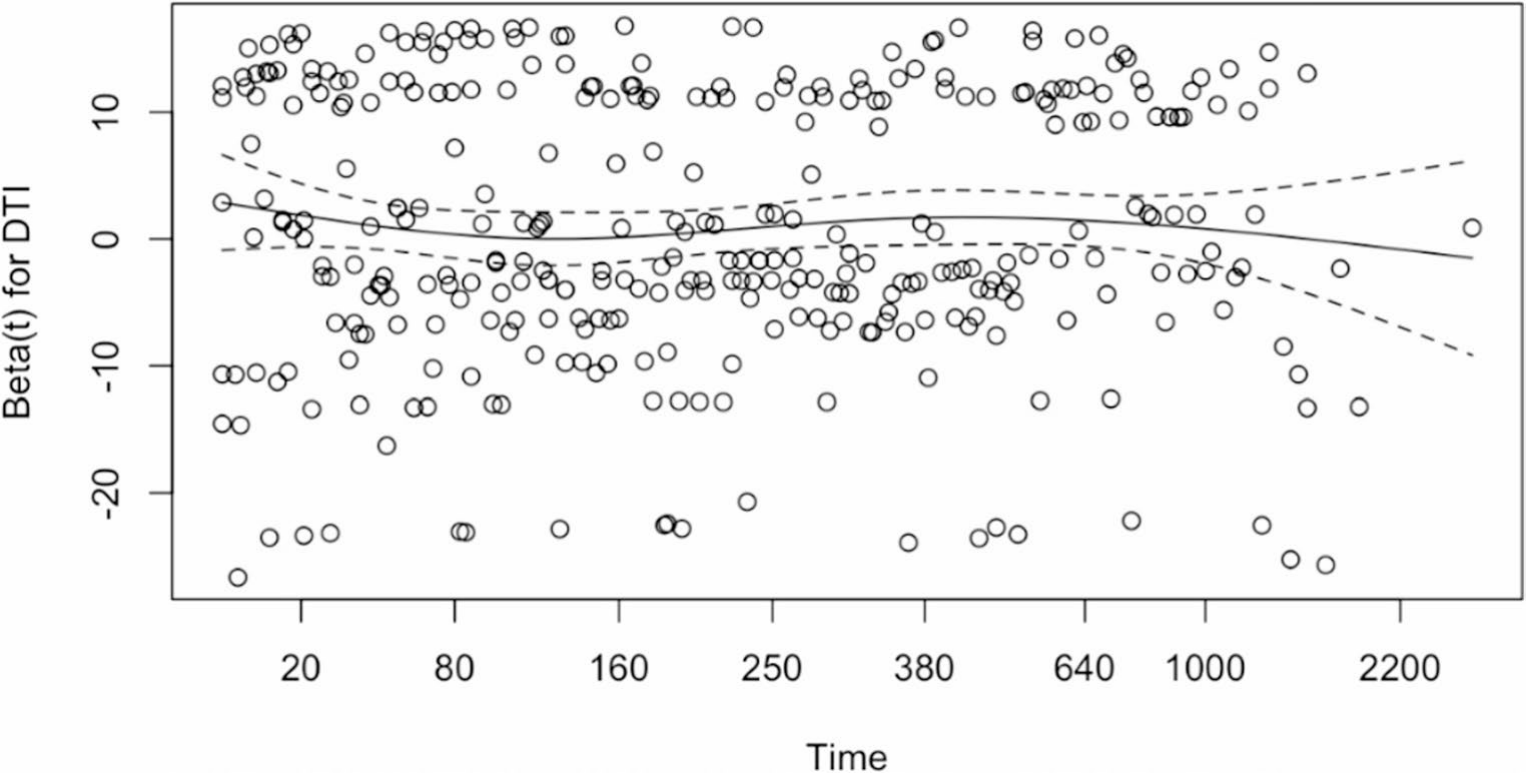
Schoenfeld residuals over time for the DTI variable, testing the proportional hazards assumption (p=0.332). The solid line represents the smoothed estimate of the time-varying coefficient B(t), with the dashed lines indicating the ± 2 standard error bounds. The non-significant p-value and random pattern of residuals around zero support the validity of the proportional hazards assumption for the primary variable of interest

The proportional hazards assumption was assessed with Schoenfeld residuals. Although the global test approached significance (*p* = 0.049), the assumption held for the DTI variable (*p* = 0.332), corroborated by residual plots showing no systematic deviation (Fig. 2).

## Discussion

Our findings reveal an unexpected association between the DTI and outcomes in older patients with AML. Specifically, prolonged DTI was associated with reduced mortality in older patients, whereas no survival benefit was observed in younger patients. Even among young patients with elevated WBC counts, treatment delay did not significantly influence outcomes. This observation differentiates our analysis from prior large registry studies, such as the German Study Alliance Leukemia–Acute Myeloid Leukemia (SAL-AML) and TriNetX registry or reports by Röllig et al. and Bertolli et al., which found no significant impact of DTI on survival [2, 3, 12].

The SAL-AML registry study by Röllig et al. reviewed 2,263 patients who received intensive induction therapy and reported a median DTI of 3 days. No association between DTI and overall survival was found (HR 1.00, *p* = 0.617). However, this study population was younger and clinically fitter than the present cohort, as all patients received intensive chemotherapy and were required to have at least 12 months of follow-up, which may have introduced survivor bias. In contrast, the current cohort likely comprises older and more frail patients treated with a wider range of therapeutic intensities, including venetoclax-based regimens.

Sekeres et al., in a cohort of 1,317 patients, reported that a DTI greater than 5 days was associated with worse outcomes in younger patients (under 60 years), with no observed impact in older patients [1]. While this finding partially corresponds to the present observation that older patients may better tolerate treatment delays, a key distinction is that the current analysis shows a paradoxical survival benefit with longer DTI in older patients, whereas Sekeres et al. found no effect in this age group.

As this cohort largely predated the widespread use of venetoclax, most patients received either intensive chemotherapy or hypomethylating agents alone. In contrast, more recent data from the COMMAND consortium, which included 488 patients with a median age of 76 years, demonstrated that a longer DTI (median 9 days) was not associated with worse survival among patients receiving HMA plus venetoclax [13]. This suggests that certain treatment regimens may influence the relationship between DTI and outcomes. Similarly, a recent study by Buzzatti et al. showed that treatment delays of up to 14days did not adversely affect overall survival or MRD negativity in intensively treated patients, also supporting the possibility of delaying therapy for molecular profiling [14].

Evaluating the effect of DTI is inherently complex due to selection bias inherent to retrospective study designs. To mitigate this bias, we stratified patients by WBC count. Interestingly, even in patients with hyperleukocytosis, prolonged DTI did not adversely influence outcomes. It is important to emphasize that these findings should not be interpreted as evidence that treatment delay is universally safe in patients with marked leukocytosis. Patients with rapidly proliferative disease, symptomatic hyperleukocytosis, leukostasis, or evolving organ dysfunction may require urgent cytoreductive therapy independent of molecular profiling results. Furthermore, patients who died within the first 10 days of diagnosis were excluded by design due to the landmark analysis, which may underestimate the true risk associated with highly proliferative or clinically unstable disease. Other potential confounders, such as disseminated intravascular coagulopathy (DIC), active infection/or sepsis, and transfusion requirements, may also have contributed to differences in early mortality and treatment tolerance.

To our knowledge, this is the first study to demonstrate a potential adverse impact of shorter DTI in older patients with AML. Several explanations may account for this finding. AML is a biologically and clinically heterogeneous disease, and older patients who received therapy earlier may have presented with greater clinical instability or more aggressive disease features, prompting expedited treatment initiation. Such patients may also have been less physiologically fit, predisposing them to treatment-related complications and early mortality. Our dataset did not capture clinical parameters such as infection or DIC, which limits our ability to test this hypothesis directly. Furthermore, our cohort largely predated the widespread use of venetoclax-based regimens, which may limit applicability to contemporary treatment paradigms [15].

An additional biologic explanation warrants consideration. Older patients with AML are more likely to harbor secondary-type or myelodysplasia-related genetic features, including TP53 mutations and other adverse-risk molecular profiles, and often present with less proliferative disease and lower white blood cell counts. As described by Senapati et al., these biologic subsets may exhibit distinct clinical behavior compared with de novo proliferative AML. In such patients, a brief delay to allow for comprehensive molecular characterization may meaningfully influence therapeutic selection [16]. For example, identification of IDH1 mutations may guide incorporation of targeted agents such as ivosidenib in combination with azacitidine, while recognition of TP53-mutated disease may influence expectations regarding benefit from venetoclax-based combinations. Although the majority of patients in our cohort were treated prior to widespread adoption of targeted agents, this biologic framework may partly explain why clinically stable older patients with lower WBC counts did not appear to be harmed by, and in some cases appeared to benefit from, longer DTI.

Given these considerations, our results should be regarded as hypothesis-generating rather than definitive evidence of causality. Notably, prior studies examining DTI in AML have employed heterogeneous definitions of “delay”, with intervals varying widely across analyses. Some modeled DTI as a continuous variable, while others used categorical thresholds, as in our study. A prior meta-analysis suggested that median DTI across studies typically ranged from four to eight days [16].

Our study has several limitations. First, its retrospective design introduces potential selection bias and confounding by indication, as patients perceived to be more acutely ill were likely prioritized for earlier treatment due to unmeasured prognostic factors. Second, the landmark analysis, while essential to minimize immortal time bias, inherently excluded patients who died within 10 days of diagnosis, thereby limiting generalizability to early survivors. Third, DTI was available as a categorical variable rather than as exact treatment initiation dates, which precluded recovery of precise treatment start dates for individual patients. As a result, overall survival was calculated from the date of AML diagnosis rather than from the start of therapy. While this approach is standard in landmark analyses, it means the pre-treatment interval contributes to the observed OS window, conferring an inherent temporal advantage to patients with longer DTI. Our landmark analysis, which anchored time zero at day 10 post-diagnosis and excluded patients who died within 10 days, was specifically designed to mitigate this immortal time bias. Importantly, baseline performance status and organ function parameters were not uniformly captured across institutions in a standardized format. Clinical instability at presentation may influence both timing of treatment initiation and early mortality risk. For example, impaired performance status due to infection may delay therapy, whereas leukemia-related organ dysfunction such as leukostasis or renal impairment may prompt urgent treatment. The absence of these variables limits our ability to fully adjust for clinical acuity and introduces potential residual confounding. Additionally, detailed mutation-level annotation, including FLT3-ITD/TKD, TP53, and RAS pathway mutations, as well as use of mutation-specific targeted therapies, were not uniformly available across centers. As such, we were unable to explore whether the association between DTI and outcomes differed across biologically defined AML subgroups. Furthermore, the majority of patients were treated in a pre-venetoclax era, which limits the applicability of our findings to fully contemporary practice. Specifically, only 152 of 698 patients (21.8%) in our cohort were treated between 2018 and 2021, the period following venetoclax’s FDA approval for AML. Given the gradual adoption of venetoclax-based regimens after approval, the true proportion of patients who received venetoclax is likely considerably smaller than this upper bound. Future studies in cohorts treated predominantly with venetoclax-based combinations will be important to determine whether the relationship between DTI and outcomes differs in the modern therapeutic landscape.

## Conclusion

In this multi-institutional retrospective study, a prolonged diagnosis-to-treatment interval was associated with improved survival among clinically stable older patients with newly diagnosed AML, particularly those with lower white blood cell counts, while no adverse effect of treatment delay was observed in younger patients. These findings challenge the traditional assumption that immediate initiation of therapy universally improves outcomes and underscore the importance of individualized treatment timing in the era of precision oncology. Prospective studies are warranted to validate these results and to determine whether a brief delay to obtain comprehensive molecular data and optimize therapeutic selection may improve outcomes in clinically stable patients.

## Supplementary Information

Below is the link to the electronic supplementary material.

Supplementary Material 1

## Data Availability

Institutional review board approval and data-sharing agreements were obtained at all participating sites. Data is saved in password protected de-identified excel sheet database.
