# Supplementary material for: Impact of diagnosis-to-treatment interval on the outcome of patients with acute myeloid leukemia
Source: Ann Hematol. 2026 May 9;105(7):312. doi: 10.1007/s00277-026-07052-7 (PMC13323866; doi:10.1007/s00277-026-07052-7)
Supplement: Supplementary file 1 — Supplementary Material 1 [file 277_2026_7052_MOESM1_ESM.pdf]

**Variables**	**Excluded patients (n=35)**
**Locations, n (%)**	
Georgetown	9 (25.7%)
GW	6 (17.1%)
University of Washington	17 (48.6%)
UVA	3 (8.6%)
**Age, median (IQR)**	71.0 (57.5, 76.0)
**WBC ($\times 10^3/\mu$L), median (IQR)**	65.9 (16.1, 123.0)
**DTI group, n (%)**	
$<$ 1 to 5 days	14 (40.0%)
6 to 10 days	2 (5.7%)
$>$ 10 days	0 (0.0%)
NA	19 (54.3%)
**ELN Risk, n (%)**	
Favorable	3 (8.6%)
Intermediate	8 (22.9%)
Adverse	16 (45.7%)
NA	8 (22.9%)
**Treatment Intensity, n (%)**	
High intensity	10 (28.6%)
Low intensity	6 (17.1%)
NA	19 (54.3%)
**Death Events, n (%)**	
Censored	5 (14.3%)
Death	30 (85.7%)

Table S1: Characteristics of patients excluded from the landmark analysis.

**Covariates**	**Hazard Ratio (HR)**	**95% CI**	**p-value**
**DTI**			
$<$ 1 to 5 days	Ref.	–	–
6 to 10 days	1.05	(0.76, 1.45)	0.773
$>$ 10 days	0.64	(0.48, 0.86)	0.004
Age (centered, per year)	1.03	(1.02, 1.04)	< 0.001
WBC (per $10^3/\mu$L)	1.002	(1.001, 1.004)	0.010
**ELN Risk**			
Favorable	Ref.	–	–
Intermediate	1.64	(1.17, 2.29)	0.004
Adverse	2.10	(1.51, 2.91)	< 0.001
**Treatment Intensity**			
High intensity	Ref.	–	–
Low intensity	1.58	(1.22, 2.05)	< 0.001

Table S2: Sensitivity analysis: Cox proportional hazards model stratified by treatment location with site-specific baseline hazards.

**Covariates**	**Hazard Ratio (HR)**	**95% CI**	**p-value**
**DTI**			
$<$ 1 to 5 days	Ref.	–	–
6 to 10 days	0.70	(0.43, 1.16)	0.165
$>$ 10 days	0.84	(0.56, 1.26)	0.395
Age $>$ 65	2.12	(1.51, 2.96)	$<$ 0.001
WBC $>$ 100	1.29	(0.85, 1.94)	0.227
**ELN Risk**			
Favorable	Ref.	–	–
Intermediate	1.70	(1.22, 2.39)	0.002
Adverse	2.18	(1.56, 3.03)	$<$ 0.001
**Treatment Intensity**			
High intensity	Ref.	–	–
Low intensity	1.72	(1.34, 2.21)	$<$ 0.001
**DTI** $\times$ **Age** $>$ 65			
6 to 10 days $\times$ Age $>$ 65	1.70	(0.91, 3.21)	0.098
$>$ 10 days $\times$ Age $>$ 65	0.73	(0.45, 1.18)	0.197
**DTI** $\times$ **WBC** $>$ 100			
6 to 10 days $\times$ WBC $>$ 100	2.45	(0.95, 6.33)	0.064
$>$ 10 days $\times$ WBC $>$ 100	0.90	(0.21, 3.91)	0.887

Table S3: Sensitivity analysis: Cox proportional hazards model with age and WBC interaction terms, stratified by treatment location.

**DTI group**	**2010–2015**	**2016–2021**
1–5 days	103 (42.4%)	221 (48.6%)
6–10 days	44 (18.1%)	73 (16.0%)
Over 10 days	96 (39.5%)	161 (35.4%)
Total	243 (100.0%)	455 (100.0%)

Table S4: Distribution of DTI groups by diagnosis period.

**Test**	**Result**
Fisher's exact test	Two-sided
Simulated p-value	0.2923
Number of replicates	200,000

Table S5: Fisher's exact test comparing DTI distributions between diagnosis periods.
